# A Two‐Tailed Phosphopeptide Crystallizes to Form a Lamellar Structure

**DOI:** 10.1002/anie.201609877

**Published:** 2017-02-13

**Authors:** Michal Pellach, Sudipta Mondal, Karl Harlos, Deni Mance, Marc Baldus, Ehud Gazit, Linda J. W. Shimon

**Affiliations:** ^1^ Department of Molecular Microbiology and BiotechnologyGeorge S. Wise Faculty of Life SciencesTel Aviv UniversityRamat Aviv69978Israel; ^2^ Division of Structural BiologyWellcome Trust Centre for Human GeneticsUniversity of OxfordRoosevelt DriveOxfordOX3 7BNUK; ^3^ NMR SpectroscopyBijvoet Center for Biomolecular ResearchUtrecht UniversityPadualaan 83584 CHUtrechtThe Netherlands; ^4^ Department of Materials Science and EngineeringIby and Aladar Fleischman Faculty of EngineeringTel Aviv UniversityRamat Aviv69978Israel; ^5^ Department of Chemical Research SupportWeizmann Institute of ScienceRehovot76100Israel

**Keywords:** membrane mimetics, peptides, self-assembly, supramolecular chemistry, X-ray crystallography

## Abstract

The crystal structure of a designed phospholipid‐inspired amphiphilic phosphopeptide at 0.8 Å resolution is presented. The phosphorylated β‐hairpin peptide crystallizes to form a lamellar structure that is stabilized by intra‐ and intermolecular hydrogen bonding, including an extended β‐sheet structure, as well as aromatic interactions. This first reported crystal structure of a two‐tailed peptidic bilayer reveals similarities in thickness to a typical phospholipid bilayer. However, water molecules interact with the phosphopeptide in the hydrophilic region of the lattice. Additionally, solid‐state NMR was used to demonstrate correlation between the crystal structure and supramolecular nanostructures. The phosphopeptide was shown to self‐assemble into semi‐elliptical nanosheets, and solid‐state NMR provides insight into the self‐assembly mechanisms. This work brings a new dimension to the structural study of biomimetic amphiphilic peptides with determination of molecular organization at the atomic level.

The self‐assembly of phospholipid bilayers in nature has inspired numerous designs of synthetic peptidic self‐assembling building blocks.^1^ Such peptide designs and careful molecular engineering have contributed a significant proportion of the study of peptide self‐assembly,^2^ with a variety of biological and non‐biological applications.^3^ However, the difficulty in crystallizing amphiphilic peptides has significantly limited the possibilities of providing an accurate depiction of the molecular organization of lipid‐like peptides.

Linear lipid‐like peptidic designs include a charged “head”, such as lysine or glutamic acid residues, and a hydrophobic “tail” comprising residues such as alanine, valine, or leucine.2a Their self‐assembly has been analyzed using microscopy, spectroscopy, X‐ray diffraction, X‐ray scattering, and neutron scattering, as well as molecular analysis using molecular dynamics simulations or solid‐state NMR (ssNMR).3b, 4 Previously designed branching amphiphilic peptides have been demonstrated, using coarse‐grained simulation, to form a self‐assembled bilayer and aqueous‐filled vesicles. The two‐tailed design was considered essential for vesicle formation, and the linear equivalent did not self‐assemble into defined structures.^5^

We recently described the design and self‐assembly of a phospholipid‐inspired phosphopeptide into semi‐elliptical nanosheets. We lacked solid evidence of the existence of a bilayer using X‐ray scattering or molecular dynamics simulations. Unique to our design is exploitation of the β‐hairpin for forming a hydrophilic head and two hydrophobic tails, with phosphorylation at the head. We also incorporated the dephenylalanine motif, which is known to facilitate self‐assembly and structure stability. The resulting sequence was Ac‐Phe‐Phe‐Val‐Lys‐(d)Pro‐pSer‐Glu‐Val‐Phe‐Phe‐NH_2_, where pSer is phosphoserine (Figure S1 in the Supporting Information).^6^ Here, we present the crystal structure of the β‐hairpin phosphopeptide, which reveals eight β‐hairpins per asymmetric unit and the formation of a bilayer. To our knowledge, this is the first example of a crystal structure of a bilayer‐forming two‐tailed peptide.

Based on previous works, the β‐hairpin β‐turn is induced by (d)Pro‐(l)Xxx,^7^ while the remaining amino acid residues form an antiparallel β‐sheet. Eight β‐hairpin peptides are observed in the asymmetric unit of the crystal structure, and the dihedral angles of all of the β‐sheet‐forming residues fall in the favored region of the Ramachandran plot (Figure 1 a and Table S1 in the Supporting Information). A close analysis of the dihedral angles of the (d)Pro 5‐(l)pSer 6 turn confirmed a type II′ β‐turn (Figure 1 a and Table S2). Superimposition of the eight molecules reveals fluctuations from a perfect β‐hairpin at the acetylated/amidated terminals (Figure 1 b). Consistent with previously designed β‐hairpin structures is the extended antiparallel β‐sheet formed by adjacent peptides (Figure 1 c),^8^ with non‐hydrogen‐bonding pairs within the β‐hairpin H‐bonding with adjacent β‐hairpins. While adjacent peptides are in the same *ac* plane, peptides that lie head‐to‐head form an additional plane that is out‐of‐register along the *b* axis. Despite this lack of alignment, the β‐hairpins that lie tail‐to‐tail appear well aligned on each *ac* plane (Figure 1 d).

**Figure 1:**
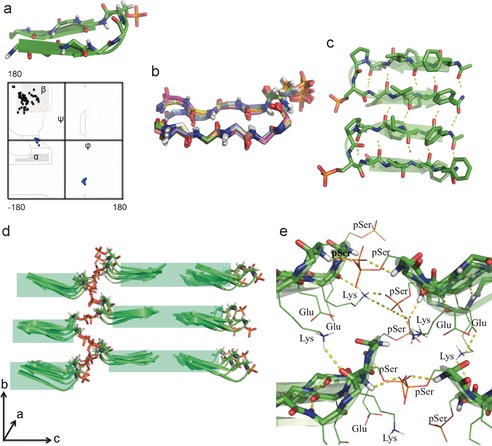
The phosphopeptide β‐hairpin structure and selected hydrogen‐bonding interactions.^16^ a) The β‐hairpin conformation of each asymmetric molecule in the crystal structure. In the Ramachandran plot, the turn angles are plotted in blue and the torsion angles of the β‐sheet‐forming residues are plotted in black. The (d)Pro‐(l)pSer dihedral angles confirm the formation of a type II′ turn. All the dihedral angles of the β‐sheet‐forming residues fall in the favored regions. b) Backbone superimposition of all of the asymmetric molecules gave an RMSD value of 0.4, which shows that all eight molecules adopt canonical β‐hairpin conformation. The phosphate moiety extends away from the hydrophobic tails. c) Intra‐ and intermolecular H‐bonding interactions of adjacent β‐hairpin peptides form a continuous antiparallel β‐sheet structure d) A shift along the *b* axis is observed for head‐to‐head interacting β‐hairpin phosphopeptides. e) Complex intra‐ and intermolecular H‐bonding interactions explain the shift along the *b* axis observed in (d), with each phosphopeptide interacting with peptides in the same *ac* plane as well as in two additional *ac* planes. Only polar side‐chain residues at the interface are displayed.

The (d)Pro 5 and (l)pSer 6 residues nucleate the turn segment of the peptide. Serine was placed within the β‐turn to allow (phospholipid‐inspired) phosphorylation at the head of our peptide, thereby creating a highly hydrophilic type II′ β‐turn. Interestingly, the phosphate groups showed little fluctuation in space, considering the possibility of free rotation around the single bonds, and remained extended away from the hydrophobic tails (Figure 1 b). This is explained by various interactions observed in the hydrophilic region of the lattice (Figure 1 e) comprising Lys 4‐(d)Pro 5‐pSer 6‐Glu 7. These interactions include: 1) intermolecular H‐bonding between phosphate groups and peptide backbone amides; 2) a lysine that forms H bonds simultaneously with a carbonyl (intramolecular) and two phosphates (intermolecular); 3) an electrostatic interaction between Lys and Glu (observed once in the crystal structure). In addition, a cluster of water molecules present at the interface H‐bond with backbone amides and side‐chain groups (Figure S2).

The hydrophobic region of the peptide comprises Phe 1‐Phe 2‐Val 3 and Val 8‐Phe 9‐Phe 10 in an antiparallel β‐sheet arrangement. The aromatic moieties both produce hydrophobicity and form intra‐ and intermolecular aromatic interactions that contribute to structure stability. Aromatic interactions occur in three *ac* planes in the crystal structure: above all eight molecules, between the two *ac* planes formed by the peptide backbones, and below all eight molecules (Figure 2 a,b). Within the upper plane, intra‐ and intermolecular edge‐to‐face aromatic interactions are continuous along the *a* axis to form a zipper‐like arrangement of the phenyl rings (Figure 2 a). These tail‐to‐tail (*c* axis) intermolecular aromatic forces appear to cause a shift along the *a* axis to give an out‐of‐register molecular arrangement. The aromatic interactions between the two *ac* planes formed by the peptide backbones occur between Phe 2 and Phe 9 of the upper peptides, and Phe 1 and Phe 10 of the peptides beneath (Figure 2 b). The electron cloud between these two planes keeps a fixed distance of approximately 1 nm between them, and comprises aromatic interactions along both the *b* and *c* (tail‐to‐tail) axes.

**Figure 2:**
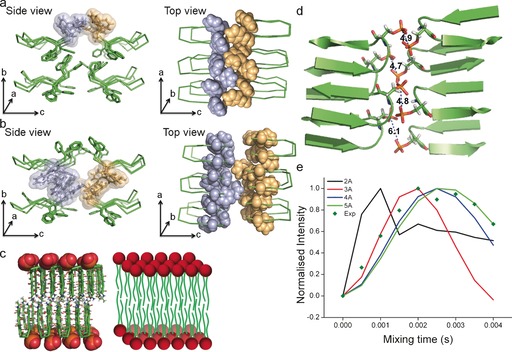
Aromatic interactions observed in three *ac* planes and the phosphopeptide bilayer.^16^ a) Aromatic interactions at the upper *ac* plane. The top view reveals a zipper‐like pattern formed by the phenyl rings, which is responsible for the shift along the *a* axis of tail‐to‐tail peptides, resulting in an out‐of‐register molecular arrangement along the *c* axis. b) Aromatic interactions at the *ac* plane between two planes formed by the peptide backbones. The intra‐ and intermolecular interactions occur along each of the three axes. c) A visual comparison between the two‐tailed phosphopeptide bilayer and a phospholipid bilayer. d) Single‐crystal analysis showing the closest unique distances between phosphate groups, averaging around 5 Å, which are repeated during crystal packing. e) Normalized ^31^P‐^31^P ssNMR double quantum build‐up curves of self‐assembled nanosheets, with the solid lines representing simulations using various ^31^P‐^31^P internuclear distances and the dark green diamonds representing experimental data points. The measured P−P distances of around 5 Å in the nanosheet structures correlate well with the approximately 5 Å found in the crystal lattice of the phosphopeptide.

Differing from previous β‐hairpin peptide crystal structures8a,8b, 9 is the pronounced separation of the peptides into “layers” to essentially form a lamellar structure (Figure 2 c). Additional previously designed amphipathic β‐hairpins with alternating hydrophilic and hydrophobic residues, which create a hydrophilic face and a hydrophobic face, stack to form face‐to‐face dimers.^10^ Their structure, structure manipulation, self‐assembly, and biological applications were mostly studied using solution and ssNMR. Further ssNMR structural study of the MAX1 peptide, which has a very similar structure, showed that such peptides stack in an *anti* (head‐above‐tail) orientation.8d Other amphipathic β‐hairpins have formed face‐to‐face dimers both in *syn* (head‐above‐head) and *anti* relative orientations, which can be affected by their environment.^11^ The phosphopeptide bilayer is comparable to that of amphiphilic peptide arrangements of KLVFF‐containing peptides, which form helical bilayer nanotapes that close to form nanotubes.^12^ The tail‐to‐tail bilayer of the phosphopeptide differs from other bilayer‐forming KLVFF‐based peptidic bilayers, which have a head‐to‐tail arrangement and interdigitated peptide ends.^13^ The stacked (parallel or antiparallel) β‐sheet arrangements and the approximately 10 Å distance between them are consistent between previous reports of peptide bilayers as well as our phosphopeptide bilayer.

In a manner similarly to that found in phospholipids, the lamellar molecular packing in our crystal (Figure 2 c) is stabilized by tail‐to‐tail hydrophobic interactions, while the charged hydrophilic heads face each other and are available for electrostatic interactions and H‐bonding. The thickness of the bilayer (ca. 4 nm) is similar to that of a typical phospholipid bilayer (Figure 2 c), as is the interdigitation of the phosphate groups.^14^ We note that the current comparisons drawn to the phospholipid bilayer are structural rather than behavioral or functional. Behavioral/functional studies have been somewhat hindered by insolubility in water, and would require structural modification to increase the water solubility.

Self‐assembly studies showed initial formation of vesicle‐like structures, which then transition into fibers and then nanosheets.^6^ Elucidation of the crystal structure enabled us to gain further insight into the molecular arrangement of the phosphopeptides within the self‐assembled semi‐elliptical nanosheets, which are formed upon switching the solvent from hexafluoroisopropanol to water. Short distances between the phosphorous atoms in the self‐assembled structures could be identified by ^31^P ssNMR using a homonuclear dipolar recoupling sequence. ssNMR analysis of the self‐assembled structures indicated distances of approximately 5 Å between phosphorus atoms, which correlates well with the distances found in the crystal structure (Figure 2 d,e). Furthermore, the out‐of‐register alignment along both the *a* and *b* axes in the crystal structure gives further insight into possible molecular arrangements responsible for the curves of the semi‐elliptical nanosheets.

Overall, we have revealed the structure of a phospholipid‐inspired phosphopeptide that crystallizes to form a bilayer. The peptides adopt an ideal β‐hairpin conformation and interact along all three axes through aromatic interactions as well as H‐bonding, including β‐sheet formation. The crystal structure and ^31^P ssNMR provide insight into the self‐assembly mechanisms and possible molecular arrangements of previously self‐assembled nanostructures. Amphiphilic peptides have demonstrated potential applications as antimicrobial agents that act through membrane disruption, cell‐culture scaffolds, drug‐delivery vehicles, and membrane‐protein stabilizers.12b, 15 The bilayer‐forming phosphopeptide (in its current form or a modified, more water‐soluble form) possesses similar potential applications, and paves the way for future exploration of its interaction with biological membranes and potential applications in biotechnology. Primarily, though, the crystal structure demonstrates the capacity of peptides to mimic the self‐assembly propensities of naturally occurring macromolecules, and could be invaluable in enhancing the predictability of intermolecular interactions for future oligopeptide designs.

The crystallographic data have been deposited in the Cambridge Structural Data Bank with CCDC no. 1502827.

## Conflict of interest

The authors declare no conflict of interest.

## Supporting information

As a service to our authors and readers, this journal provides supporting information supplied by the authors. Such materials are peer reviewed and may be re‐organized for online delivery, but are not copy‐edited or typeset. Technical support issues arising from supporting information (other than missing files) should be addressed to the authors.

Supplementary
