# Supplementary material for: A Two‐Tailed Phosphopeptide Crystallizes to Form a Lamellar Structure
Source: Angew Chem Int Ed Engl. 2017 Feb 13;56(12):3252–5. doi: 10.1002/anie.201609877 (PMC5412914; doi:10.1002/anie.201609877)
Supplement: Supplementary file 1 — Supplementary [file ANIE-56-3252-s001.pdf]

Angewandte Chemie
Eine Zeitschrift der Gesellschaft Deutscher Chemiker

Supporting Information

A Two-Tailed Phosphopeptide Crystallizes to Form a Lamellar Structure

Michal Pellach[+], Sudipta Mondal[+], Karl Harlos, Deni Mance, Marc Baldus, Ehud Gazit, and Linda J. W. Shimon**

anie_201609877_sm_miscellaneous_information.pdf

A two-tailed phosphopeptide crystallises to form a lamellar structure

Supporting information

Experimental section:
X-ray crystallography
^{31}P ssNMR of self-assembled nanosheets.

Figures:
Figure S1. Designed phospholipid-inspired β-hairpin phosphopeptide.
Figure S2. Hydrogen bonding interactions between the phosphopeptide and water.

Tables:
Table 1. Torsion angles of Type II' β turn
Table 2. Torsion angles of β-sheet forming residues
Table 3. Crystal data and structure refinement for the peptide crystal

Experimental Section

X-ray crystallography

Crystal preparation and data collection. The peptide was screened for crystallization conditions using different ratios of trifluoroethanol, acetonitrile and water. Crystals used for data collection were grown in Linbro-type crystallisation plates by vapour diffusion. The dry peptide was first dissolved at a concentration of 16 mg/mL in trifluoroethanol, then the peptide solution was mixed in a ratio of 1:1 (v/v) with a reservoir solution containing trifluoroethanol / acetonitrile / water 2:1:1 (v/v/v) and then equilibrated as sitting drops against the reservoir solution. Starting drop volumes varied between 4 and 24 μl. Plate-like crystals grew within 2-3 days at room temperature (21°C). Once fully grown crystals were moved to 5°C as they tended to degrade when left at room temperature. For data collection crystals were picked up in a cryo-loop, transferred to a solution containing 75% reservoir and 25% glycerol (v/v) and then frozen by plunging them into liquid nitrogen. Diffraction data were collected at 100 K at the Diamond Light Source synchrotron (Didcot, UK), beam-line I03 using a Pilatus3 6M detector and a wavelength of 0.70Å. A single sweep of 7200 images was collected as 0.1° frames to a detector resolution of 0.82Å (0.71Å in corners). The data were auto-processed using xia2 and 3dii.[1]

Processing and structural refinement of crystal data. The structure was solved by direct methods in SHELXT-2013.[2] Six of the eight independent peptide molecules were clearly visible, while two additional molecules were partially seen. The missing atoms were added by alternating cycles of refinement in SHELXL-2013 using manual inspection of the 2Fo-Fc and Fo-Fc electron density maps in Coot.[3] Alternate conformations of the peptide side chains and solvent molecules were added where clearly visible. The refinements in SHELXL-2013 were weighted full-matrix least-squares against $|F^2|$ using all data. In the final stages of refinement SQUEEZE[4] protocol

was used due to the large voids and remaining disordered solvent molecules. Atoms were refined independently and non-solvent atoms were refined anisotropically with the exception of hydrogen atoms, which were placed in calculated positions and refined in a riding mode. Crystal data collection and refinement parameters are given in Supplementary Table 3 and the complete data can be found in the cif file as supplementary information. The crystallographic data have been deposited in the CCDC with no. 1502827.

^{31}P ssNMR of self-assembled nanosheets.

The phosphopeptide was dissolved in hexafluoroisopropanol (100 mg/mL) and diluted with water to give a final concentration of 5mg/mL. Self-assembled nanosheets were allowed to form at room temperature over several hours, as previously described.[5] The nanosheets were centrifuged in a rotor and excess moisture was removed. ^{31}P-NMR measurements were performed using a wide-bore 500MHz (Bruker Biospin) system with a 3.2-mm magic-angle-spinning (MAS) probe head. The sample was spun at an MAS frequency of 8kHz and the measurements were performed at an effective temperature of 280 K. After direct excitation of the ^{31}P nuclei SPC5[6] dipolar recoupling was used for generating double quantum coherences. For the measurements the relaxation delay was set to 3 seconds. A ^{1}H field strength of 83.3 kHz was used for decoupling and a field strength of 71.4 kHz was use for the direct excitation pulse on ^{31}P. Simulations of the double-quantum buildup curves were performed using SIMPSON[7] for various ^{31}P-^{31}P distances. Span (δ_{CSA}) and the asymmetry parameter (η_{CSA}) of the chemical shift anisotropy used in our simulations were determined experimentally from an MAS sideband analysis and set to 49 ppm and 0.71, respectively.

Figures

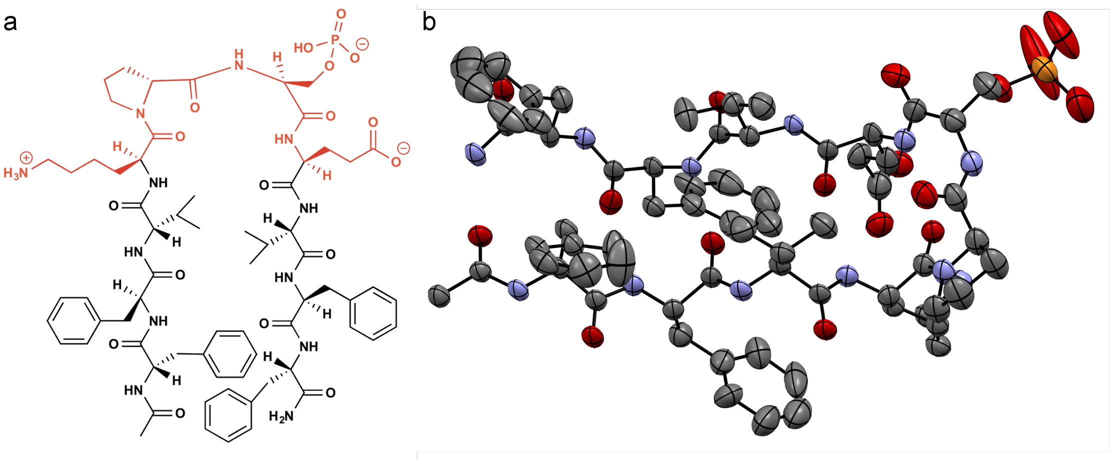

Figure S1. Designed phospholipid-inspired β-hairpin phosphopeptide. (a) Chemical structure. The hydrophilic region of the designed peptide (red) is composed of four residues: lysine, (D)proline, phosphoserine and glutamic acid. The phosphorylated hydrophilic “head” resembles the “head” of a phospholipid. The well-studied diphenylalanine motif is incorporated into each of the hydrophobic tails to facilitate self-assembly via aromatic interactions. (b) Oak Ridge Thermal Ellipsoid Plot (ORTEP) of a molecule from the crystal structure analysis. H's and solvents are omitted for clarity. The ORTEP is drawn at 50% probability level.

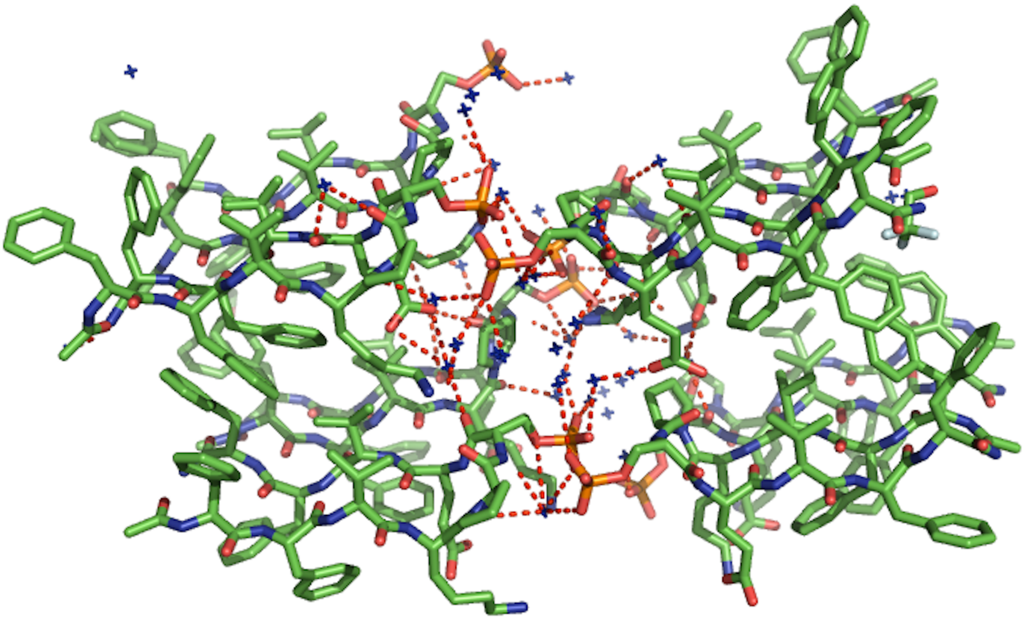

Figure S2. Hydrogen bonding interactions between the phosphopeptide and water in the crystal structure. Several water molecules are found in the hydrophilic region of the crystal structure. While acetic acid solvent molecules have been observed in the hydrophilic region of a phospholipid crystal,[8] observation of water molecules is rare. In the phosphopeptide bilayer, water molecules between the phosphopeptide heads appear to assist in stabilisation of the lamellar structure. Hydrogen bonding interactions with water are mainly found between water and the phosphate groups, though are also observed between water and the lysine and glutamic acid residues.

Table 1. Torsion angles of β-sheet forming residues.

φ	ψ
-127.08	94.74
-128.41	127.72
-141.30	140.35
-88.51	126.16
-147.17	86.32
-132.26	140.98
-128.86	133.34
-125.19	123.39
-137.80	131.50
-126.10	137.10
-132.70	131.20
-82.70	119.70
-127.60	93.10
-134.09	123.80
-128.20	126.10
-111.80	146.20
-121.50	112.90
-126.00	138.90
-130.90	137.30
-95.10	137.30
-127.90	84.70
-119.20	116.50
-129.10	126.70
-103.10	118.20
-135.90	117.80
-117.20	120.00
-129.70	135.50
-91.00	136.60
-116.10	96.60
-120.30	123.80
-142.90	137.00
-159.20	165.70

φ	Ψ
-124.10	128.50
-122.50	130.80
-143.40	134.00
-83.60	126.80
-142.80	97.60
-128.20	129.20
-122.00	122.90
-105.00	140.50
-132.00	112.60
-115.90	120.60
-125.70	130.70
-99.70	136.70
-124.40	102.40
-123.30	124.00
-144.10	145.30
-162.60	166.20
-118.10	115.30
-133.40	123.50
-138.90	137.10
-97.30	156.90
-128.00	101.40
-117.50	121.00
-120.80	126.90
-99.50	112.90
-119.50	95.00
-125.30	126.90
-141.70	134.90
-86.30	131.10
-136.20	101.40
-126.20	128.10
-139.50	147.20
-126.40	130.60

Table 2: Torsion angles of Type II' β turn: residues (D)Pro5-pSer6 .

Asymmetric unit	Residues	φ	ψ
1	Pro	62.6	-135.9
	pSer	-84.7	4.2
2	Pro	65.8	-119.0
	pSer	-81.1	-15.1
3	Pro	60.8	-133.0
	pSer	-80.8	0.7
4	Pro	55.0	-127.4
	pSer	-88.3	3.8
5	Pro	64.1	-120.2
	pSer	-87.8	-5.4
6	Pro	59.6	-122.1
	pSer	-86.2	-6.5
7	Pro	61.7	-127.1
	pSer	-92.8	5.5
8	Pro	57.8	-129.5
	pSer	-90.9	4.6

Table 3. Crystal data and structure refinement for the peptide crystal.

Empirical formula	$4(C_{67}H_{89}N_{12}O_{17}P)$, $C_2H_3F_3O$, 18(O)
Formula weight	5849.95
Crystal system	Triclinic
Space group	*P*1
a, Å	19.050(4)
b,Å	21.750(4)
c, Å	39.540(8)
α°	97.62(3)
β°	94.07(3)
γ°	91.31(3)
V (Å^3)	16189(6)
Z	2
dcalc (mg/cm^3)	1.200
μ (mm^{-1})	0.110
Reflections	189390
Unique reflections	70256
R_{merge}	0.035
R [I > 2 σ (I)]	R_1 = 0.1178, wR_2 = 0.3240
Goodness of Fit	1.537

$R_1 = \Sigma||F_o| - |F_c||/\Sigma|F_o|$; $wR_2 = \{\Sigma [w(F_o^2 - F_c^2)^2]/\Sigma w(F_o^2)^2]\}^{1/2}$

References

[1] a) Collaborative Computational Project, *Acta Crystallogr. Sect. D-Biol. Crystallogr.* **1994**, *50*, 760-763; b) P. Evans, *Acta Crystallogr. Sect. D-Biol. Crystallogr.* **2006**, *62*, 72-82; c) W. Kabsch, *Acta Crystallogr. Sect. D-Biol. Crystallogr.* **2010**, *66*, 125-132; d) N. K. Sauter, R. W. Grosse-Kunstleve, P. D. Adams, *J. Appl. Crystallogr.* **2004**, *37*, 399-409; e) G. Winter, *J. Appl. Crystallogr.* **2010**, *43*, 186-190; f) Z. Zhang, N. K. Sauter, H. van den Bedem, G. Snell, A. M. Deacon, *J. Appl. Crystallogr.* **2006**, *39*, 112-119.

[2] G. Sheldrick, University of Göttingen, Göttingen, Germany, 2013.

[3] P. Emsley, K. Cowtan, *Acta Crystallogr. Sect. D-Biol. Crystallogr.* **2004**, *60*, 2126-2132.

[4] A. Spek, *Acta Crystallogr. Sect. D-Biol. Crystallogr.* **2009**, *65*, 148-155.

[5] M. Pellach, Y. Atsmon-Raz, E. Simonovsky, H. Gottlieb, G. Jacoby, R. Beck, L. Adler-Abramovich, Y. Miller, E. Gazit, *ACS Nano* **2015**, *9*, 4085-4095.

[6] M. Hohwy, C. M. Rienstra, C. P. Jaroniec, R. G. Griffin, *J. Chem. Phys.* **1999**, *110*, 7983-7992.

[7] M. Bak, J. T. Rasmussen, N. C. Nielsen, *J. Magn. Reson.* **2000**, *147*, 296-330.

[8] P. B. Hitchcock, R. Mason, K. M. Thomas, G. G. Shipley, *Proc. Natl. Acad. Sci. U. S. A.* **1974**, *71*, 3036-3040.
